# Early post-transplant serum IgA level is associated with IgA nephropathy recurrence after kidney transplantation

**DOI:** 10.1371/journal.pone.0196101

**Published:** 2018-04-25

**Authors:** Anne-Sophie Garnier, Agnès Duveau, Julien Demiselle, Anne Croué, Jean-François Subra, Johnny Sayegh, Jean-François Augusto

**Affiliations:** 1 LUNAM Université, Angers, France; 2 Service de Néphrologie-Dialyse-Transplantation, CHU Angers, Angers, France; 3 Département de pathologie cellulaire et tissulaire, CHU Angers, Angers, France; INSERM1163, FRANCE

## Abstract

IgA nephropathy (IgAN), the most frequent primary glomerulonephritis, affects young patients and is associated with a high risk of progression to end-stage renal disease. Consequently, patients with IgAN constitute an important proportion of candidates for kidney transplantation. Several studies showed a significant risk of IgAN recurrence on kidney graft, but the risks factors for recurrence remain to be accurately evaluated. Indeed, early identification of at risk patients may allow the optimization of treatment and the reduction of recurrence rate on the graft. In the present work, we studied the relationship between post-transplant serum IgA (sIgA) levels and the risk of IgAN recurrence after kidney transplantation. Recipients with IgAN had higher levels of sIgA as compared to patients with other nephropathies (p<0.05). The prevalence of IgAN recurrence was 20.8% during the period of analysis (mean follow-up of 6 ± 3.2 years). Serum IgA levels at M6, M12 and M24 post-transplant were significantly higher in patients with IgAN recurrence as compared to those without (p = 0.009, p = 0.035 and p = 0.029, respectively). Using receiver operating curve (ROC), sIgA at M6 and M12 post-transplant were significantly associated with IgAN recurrence (AUC = 0.771, p = 0.004 and AUC = 0.767, p = 0.016, respectively), while serum creatinine and proteinuria were not. Serum IgA level at month 6 was significantly associated with the occurrence of post-transplant IgA recurrence, whether it was analyzed as a continuous or a categorical variable. After successive adjustment on age, gender and proteinuria, sIgA remained a significant risk factor of post-transplant IgAN recurrence. Finally, survival free of IgAN recurrence was significantly better in patients with sIgA<222 mg/dL at month 6 as compare to IgAN patients with sIgA≥222 mg/dL (p = 0.03). Thus, the present work supports a link between post-transplant sIgA levels and IgAN recurrence and suggests that sIgA may be a valuable predictive biomarker of IgAN recurrence in kidney transplant recipients.

## Introduction

IgA nephropathy (IgAN), the most frequent primary glomerulonephritis, is characterized by immune deposition of polymeric IgA in the glomerular mesangium [1]. Clinical presentations of IgAN are heterogeneous, going from macroscopic hematuria with or without acute kidney disease to microscopic hematuria with or without proteinuria and hypertension [2]. A large proportion of patients progress to end-stage renal disease [3]. Several studies showed a high incidence of IgAN recurrence on kidney graft at variable rates, depending on length of post-transplant follow-up and indication for biopsy [4]. The diagnosis of IgAN recurrence associates a compatible clinical context (proteinuria, macroscopic or microscopic hematuria and/or kidney failure) and histological criteria, mainly the presence of mesangial IgA deposition on the graft biopsy. Diagnosis of IgAN recurrence is of particular importance because it is associated with an increased risk of graft loss, and because its diagnosis may result in therapeutic modifications [5]. Thereby, identification of biomarkers to predict IgAN recurrence on kidney graft are needed to identify at risk patients. Such biomarkers may allow optimizing treatment and decreasing the rate of IgAN recurrence.

Several studies, with retrospective designs, have evaluated the risk factors of IgAN recurrence after kidney transplantation. Known risk factors are younger age at transplantation [6–7], degree of proteinuria [7] and rapid progression of native IgAN [8]. Living donor graft [6,9] and pre-existent IgA deposits from donor [10] have also been identified as donor-related risk factors of IgAN recurrence.

In a recent study, a relationship between altered glycosylated IgA, soluble CD89 complexes and post-transplant recurrence of IgAN was identified, suggesting that IgA-soluble CD89 complexes could be a predictive biomarker of IgAN recurrence [11]. However, to date, no study has directly evaluated the relationship between post-transplant total serum IgA (sIgA) level and the risk of IgAN recurrence.

In our center, determination of immunoglobulin (Ig) classes is part of the standard prospective monitoring of kidney transplant recipients. We therefore undertook the present study to analyze the relationship between post-transplant sIgA levels and IgAN recurrence after kidney transplantation.

## Patients and methods

### Study population and study design

We conducted a retrospective cohort study approved by the local Ethics committee of Angers University Hospital (agreement 2014–84, approved on october, 2014). Given the retrospective nature of the research, patients did not provide written consent, although verbal consent was required by the Ethic Committee for kidney transplant patients with IgA nephropathy. Verbal consent was obtained from patients during their standard medical follow-up in the department. All consecutive patients who underwent kidney transplantation in Angers University Hospital between January 2003 and December 2013 (n = 471) were considered for inclusion in the study. Patients were followed until graft loss or death. Patients with missing data at the time of transplantation were excluded. Data allowing the characterization of the study population were retrieved from the department’s database and the French Transplantation database (Cristal, Agence de la Biomedicine). None of the transplant donors were from a vulnerable population and all donors or next of kin provided written informed consent that was freely given.

We collected the following clinical parameters: gender, age, weight, body mass index (BMI), treatments including immunosuppressive regimens and antihypertensive medications. Hypertension was defined as systolic blood pressure (SBP) >140 mmHg and/or diastolic blood pressure (DBP) >90 mmHg or treatment with antihypertensive drugs. We recorded the following biological parameters: 24 h urinary protein excretion, serum albumin (sAlb), serum creatinine (Scr), hemoglobin (Hb) and levels of serum Ig (IgA, IgG, IgM). Glomerular Filtration Rate (GFR) was estimated using the Modification of Diet in Renal Disease equation (MDRD) [12]. Ig levels were determined prospectively by the nephelometric technique, using a BN Prospect Nephelometer Analyser and commercially available kits from Siemens Dade Behring (Marburg, Germany).

### IgA nephropathy diagnosis

All included patients had IgA nephropathy diagnosed at kidney biopsy of native kidney. Full kidney biopsy report was available in 53/67 patients. To assess the severity of IgA nephropathy presentation on native kidney, pathology reports were reviewed by two senior nephrologists. The following lesions were classified as present or absent at biopsy: mesangial hypercellularity, endocapillary proliferation, segmental glomerulosclerosis, extracapillary proliferation, interstitial fibrosis (0, absence; 1, moderate; 2, severe), nephroangiosclerosis (0, absence; 1, moderate; 2, severe). We also analyzed the delay between IgAN diagnosis and RRT initiation, as well as the use of immunosuppressive drugs at diagnosis.

### Definition of IgAN recurrence

Both clinical and histological parameters were considered to establish the diagnosis of IgAN recurrence. Clinically, IgAN recurrence was suspected in patients with microscopic hematuria and new or worsening proteinuria and/or allograft dysfunction (defined by a significant increase of serum creatinine). Renal allograft biopsies were performed for clinical indications, including allograft dysfunction, proteinuria, and/or hematuria. All biopsies were reviewed by a senior renal pathologist. Immunofluorescence staining for IgA deposition was performed in all cases. Histological diagnosis of recurrent IgAN was defined by mesangial IgA deposition as the predominant or co-dominant immunoglobulin.

### Statistical analysis

Continuous variables were expressed as a mean ± SD and categorical variables as absolute number and percentage. Categorical and continuous data were analyzed with χ^2^ or Fischer’s exact test and Mann-Whitney U tests, respectively. The predictive values of sIgA, serum creatinine and proteinuria at month 6 and month 12 post-transplant for IgAN recurrence were analyzed using receiver operating characteristics (ROC) curves. Subsequently, a cut-off value of sIgA was determined based on the Youden index from the ROC curve at month 6 post-transplant. Univariate and multivariate logistic regression were used to analyze the risk factors associated with the presence of post-transplant IgAN recurrence. Associations are given as odds ratios (ORs) with 95% confidence intervals. Kaplan-Meyer method was used to analyze free survival of IgAN recurrence. A log-rank test was used to compare the survival curves. All *p* values were two-sided. A *p* value lower than 0.05 was considered statistically significant. Statistical analysis was performed using Graphpad Prism^®^ and SPSS software^®^ 22.0.

## Results

### Patients with IgA nephropathy have higher levels of post-transplant serum IgA than recipients with other types of nephropathies

**Fig 1:**
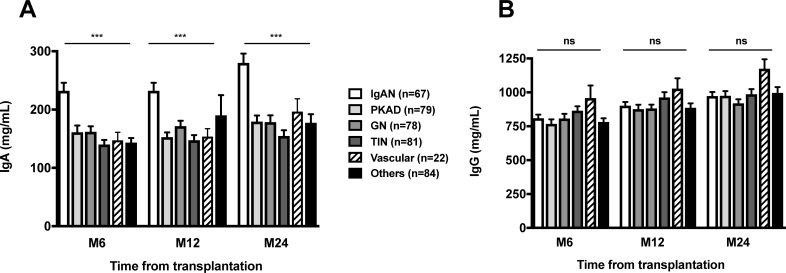
Posttransplant serum IgA (A) and IgG (B) levels at months 6, 12 and 24 from transplantation according to original nephropathy. Statistics were done using one-way anova. ***, p<0.001.

During the analysis period, 471 patients received a kidney graft. Among the 411 patients who had full clinical data and had Ig class determination, 70 patients had IgAN and were considered for the study. Three patients were excluded, 2 patients with a follow-up shorter than 6 months and 1 patient who died before 6-months of follow-up (S1 Fig). The mean follow-up of the cohort was 5.9 ± 3.2 years. At 6, 12 and 24 months after transplantation, recipients with IgAN had higher sIgA levels as compared to patients with other types of nephropathies (p<0.05) (Fig 1A), whereas serum IgG levels were not statistically different between groups (Fig 1B). Furthermore, we observed that sIgA levels increased significantly between M6 and M24 post-transplant in patients with IgAN (p<0.05, data not shown).

### Characteristics and outcomes of patients with IgA nephropathy

Donor and recipient characteristics are detailed in Table 1. Patients with previous transplantation accounted for 19.4% of cases. Grafts were from deceased donors in 92.5% of cases and 73.4% of recipients were on dialysis before transplantation. Most patients received induction therapy with basiliximab or anti-thymocyte globulins. Initial maintenance regimen associated tacrolimus and an antimetabolite (mycophenolate mofetil or mycophenolic acid) in 73.1% of cases.

**Table 1:** Characteristics and outcomes of IgA nephropathy patients.

	All patients	No IgAN Recurrence (n = 53)	IgAN Recurrence (n = 14)	*p*
	(n = 67)			
**Baseline characteristics**				
Sex (M/F)	55/12	41/12	13/1	0.272
Age (years)	41.1 ± 13.5	42.5 ± 12.8	36.1 ± 15.2	0.117
Weigh (kg)	72.3 ± 13.1	73.2 ± 13.0	69.0 ± 13.5	0.286
BMI (Kg/m^2^)	24.5 ± 4.2	25.0 ± 4.3	22.9 ± 3.5	0.109
**History of original IgA nephropathy**				
Delay between IgAN diagnosis and RRT (years)	11.0 ± 17.2	12.25 ± 18.7	6.35 ± 8.30	0.258
Histological features (n)*	53	41	12	
Mesangial hypercellularity	50 (94.3)	38 (92.7)	12 (100)	0.460
Endocapillary proliferation	44 (83.0)	33 (80.5)	11 (91.7)	0.498
Segmental glomerulosclerosis	49 (92.5)	39 (95.1)	10 (83.3)	0.308
Extracapillar proliferation	31 (58.5)	24 (58.5)	7 (58.3)	0.886
Interstitial fibrosis (0/1/2)	9/15/29	5/13/23	4/2/6	0.288
Nephroangiosclerosis (0/1/2)	18/16/19	14/12/15	4/4/4	0.924
Immunosuppressive Treatment	13 (19.4)	12 (29.3)	1 (7.7)	0.272
**History of transplantation**				
Previous transplantation	13 (19.4)	10 (18.9)	3 (21.4)	1.000
Pre-transplant dialysis	49 (73.1)	39 (73.6)	10 (71.4)	1.000
Donor age	40.0 ± 16.7	40.2 ± 15.4	39.1 ± 21.8	0.837
Type of donor				
Deceased/others	62/5	49/4	13/1	1.000
Cold ischemia time (hours)	17.4 ± 7.1	17.4 ± 6.8	17.2 ± 8.6	0.947
HLA mismatch				
HLA A&B	2.77 ± 0.11	2.75 ± 0.12	2.85 ± 0.31	0.717
HLA DR	1.19 ± 0.09	1.21 ± 0.10	1.07± 0.19	0.526
Delayed graft function	7 (10.4)	6 (11.3)	1 (7.1)	1.000
**Immunosuppressive regimens**				
Induction therapy				
None	13 (19.4)	9 (17.0)	4 (28.6)	0.446
Basiliximab	27 (40.3)	23 (43.4)	4 (28.6)	0.372
Antithymocyte globulins	27 (40.3)	21 (39.6)	6 (42.9)	0.826
Maintenance regimen				
Tacrolimus-based	49 (73.1)	38 (71.7)	11 (78.6)	0.743
Cyclosporine-based	17 (25.4)	14 (26.4)	3 (21.4)	1.000
Other	1 (1.5)	1 (1.9)	0 (0.0)	**/**
**Month 6 posttransplant biology**				
Serum creatinine	127.7 ± 41.8	126.4 ± 43.8	132.0 ± 35.4	0.340
Proteinuria	0.32 ± 1.2	0,14 ± 0.19	0.96 ± 2.6	0.471
Serum IgA level	232.2 ± 112	216.7 ± 110	290.9 ± 106	**0.027**
**Month 6 immunosuppressive treatment**				
CNI	67 (100)	53	14	
Steroids (yes/no)	27 (40.3)	22/31	5/9	0.694
Mean dose, g/day	12.5 ± 3.1	13.7 ± 3.8	7.0 ± 1.46	0.418

* Full pathological report was available for 53/67 patients.

Risk factors for IgAN recurrence were analysed using univariate analysis according to IgAN recurrence or not over the study period. IgAN recurrence was diagnosed in 14 recipients (20.8%) with a median time to recurrence of 3.13±2.0 [0.5–5.5] years. The recipient age at transplantation tended to be lower in the recurrence group (36.1±15.2 versus 42.5±12.8 years, p = 0.117). Gender distribution was comparable between groups and there was no significant difference between groups according to donor type, cold ischemia time, delayed graft function and immunosuppressive regimen. Furthermore, serum creatinine and proteinuria levels at M6 post-transplant were not significantly different whether patients experienced or not IgAN recurrence. However, sIgA level at M6 post-transplant was significantly higher in the recurrence group as compared to the group without IgAN recurrence (298.6 ± 104 vs 243.3±121, p = 0.038) (Table 1).

We did not observe any difference between patients with or without IgAN recurrence according to characteristic of IgA nephropathy presentation on native kidney. Indeed, histological features, although analysis was limited to 53/67 patients, were not statistically different. Also, there was no difference according to delay between disease onset and RRT and immunosuppressive treatment at IgAN diagnosis (Table 1).

Mean follow-up was comparable between groups. There was no significant difference in immunosuppressive treatment at month 6 posttransplant, as well as of serum creatinine at month 12 post-transplant between the two groups. At last follow-up, renal allograft function was not statistically different between groups, although lower in the IgAN recurrence group (eGFR = 46±35.4 vs 53.1±24.7 mL/min/1.73m^2^, p = 0.389). However, more patients tended to have more than 25% decrease in eGFR between months 6 and last follow-up and higher serum creatinine at last follow-up (p = 0.078 and p = 0.09, respectively). Acute rejection and graft loss rate were not significantly different between groups. These data are summarized in Table 2. Graft survival was not significantly different according to recurrence status (S2 Fig). IgAN recurrence were treated with ACEI/ARB initiation in 8 patients and oral steroids in 6 patients.

**Table 2:** Outcomes of patients according to IgA nephropathy recurrence.

	No recurrence (n = 53)	Recurrence (n = 14)	*P*
Mean follow-up	5.99 ± 3.2	5.96 ± 3.2	0.979
Acute rejection, n (%)	15 (28.3)	2 (14.3)	0.723
Serum creatinine at year 1 (μmol/L)	137.2 ± 52.4	138.0 ± 38.9	0.963
Serum creatinine at last follow-up (μmol/L)	177.7± 173	302.7 ± 306	0.09
GFR at year 1 (mL/min/1.73m2)*	54.4 ± 20.1	55.4 ± 15.6	0.861
GFR at last follow-up (mL/min/1.73m^2^)**	53.1 ± 24.7	46.0 ± 35.4	0.389
More than 25% GFR decline*, n (%)	13 (24.5)	7 (53.9%)	0.078
Graft loss, n	5 (10.4)	3 (21.4)	0.349
Death, n	0	0	/

* Patients that started renal replacement therapy were considered as having an eGFR of 5 mL/min/1.73m^2^.

^**^ Between month 6 and last follow-up.

### IgA serum level is associated with IgAN recurrence after transplantation

**Fig 2:**
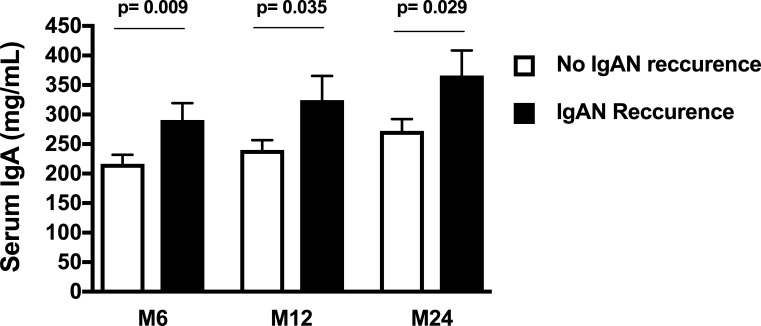
Serum IgA level at months 6, 12 and 24 posttransplant according to IgAN recurrence.

Serum IgA levels at M6, M12 and M24 post-transplant were significantly higher in patients with IgAN recurrence as compared to patients without recurrence (p = 0.01, p = 0.04 and p = 0.03, respectively) (Fig 2).

**Fig 3:**
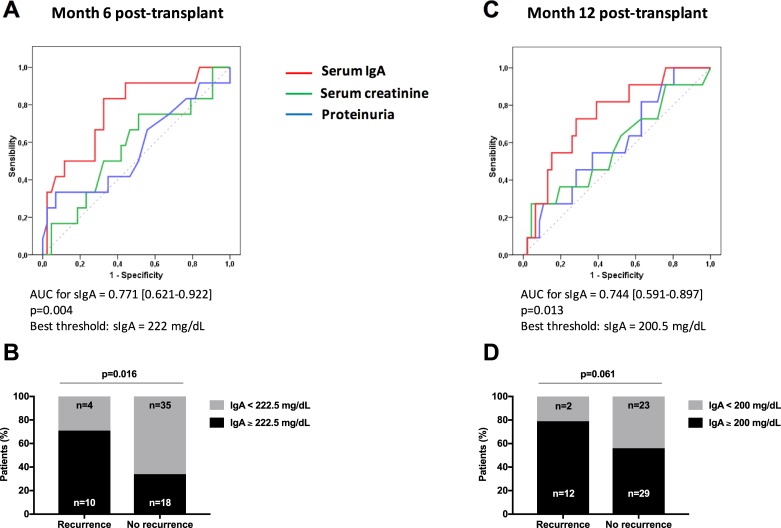
Predictive value of sIgA at month 6 and month 12 post-transplant for IgAN recurrence. ROC curve of serum IgA, proteinuria and serum creatinine at month 6 (A) and and month 12 (C) posttransplant for IgAN recurrence. Percentage of patients with sIgA above determined threshold using ROC analysis in patients with and without IgAN recurrence at month 6 (B) and month 12 post-transplant (D).

**Fig 4:**
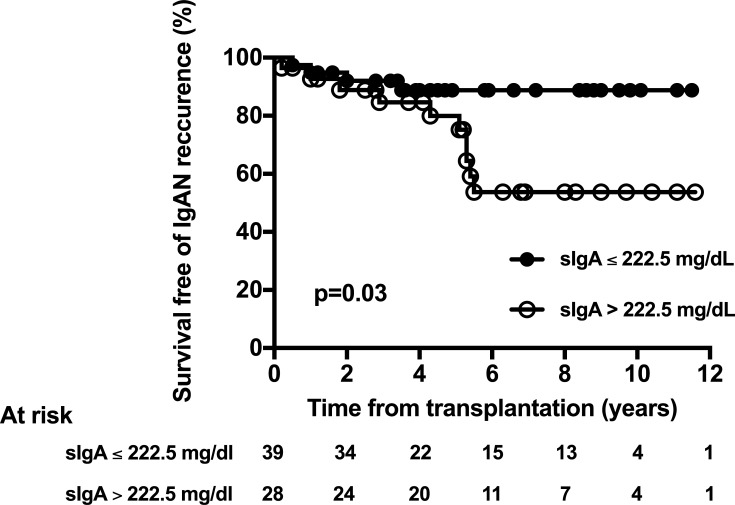
Survival free of IgA nephropathy recurrence according to serum IgA level at month 6 post-transplant. IgA threshold was previously determined using ROC curve analysis.

We next used ROC to analyze the predicting values of sIgA, serum creatinine and proteinuria for IgAN recurrence (Fig 3). Serum IgA levels at month 6 (Fig 3A) and month 12 post-transplant (Fig 3B), as assessed by the AUC, were significantly associated with IgAN recurrence (p = 0.004 and p = 0.013, respectively), while serum creatinine and proteinuria were not. According to ROC analysis, a sIgA value of 222.5 mg/dL at month 6 post-transplant appeared as the best threshold to predict IgAN recurrence (sensibility 71.4%, specificity 66.0%, predictive positive value 35.7%, negative predictive value 89.7%). Using univariate logistic analysis, sIgA was significantly associated with the occurrence of post-transplant IgAN recurrence, whatever sIgA was considered as a continuous or a categorical variable (Table 3). After successive adjustment on age or proteinuria, sIgA remained a significant risk factor of post-transplant IgAN recurrence in the multivariate analysis (Table 3). In a last step, we analyzed survival-free of IgAN recurrence according to the presence or not of a serum IgA level above 222.5 mg/dL. Survival free of IgAN recurrence was significantly greater in patients who had sIgA level below 222.5 mg/dL, as compared to patients who had sIgA above 222.5 mg/dL (Fig 4). We did not observe any evident relation between IgA level and histological severity of IgAN. However, this analysis was limited by the low number of patients with IgAN recurrence in our study.

**Table 3:** Univariate and multivariate analysis of risk factors of IgAN recurrence. In the multivariate analysis, sIgA was considered as a continuous variable or a categorical variable and successive adjustments on age and proteinuria level were done. The indicated p-value refers to sIgA.

	Association between serum IgA level and recurrence of IgAN		
	OR	95% CI	*P*
**Univariate analysis**			
Serum IgA as a continuous variable*	1.005	1.000–1.010	**0.043**
Serum IgA as a categorical variable**	5.278	1.336–17.68	**0.016**
**Multivariate analysis**			
Serum IgA as a continuous variable*			
Adjusted for:			
Age	1.007	1.001–1.012	**0.016**
Proteinuria (month 6)	1.012	1.004–1.020	**0.004**
Serum IgA as a categorical variable**			
Adjusted for:			
Age	5.981	1.536–23.29	**0.010**
Proteinuria (month 6)	15.19	2.026–113.9	**0.008**

* Per each unit increament (mg/dL).

** Serum IgA > 222.5 mg/dL.

## Discussion

In this study, we analyzed the relationship between sIgA levels and the occurrence of IgAN recurrence after kidney transplantation. Our results support a link between sIgA and the risk of post-transplant IgAN recurrence. Indeed, sIgA at month 6 post-transplant appeared as a risk factor of IgAN recurrence, independently of age and serum creatinine at month 6 post-transplant. Moreover, survival free of IgAN recurrence was significantly higher in patients who had sIgA levels above 222.5 mg/dL at month 6 post-transplant, compared to other patients.

The incidence of post-transplant recurrence of IgAN in our cohort was 20.8% after 6 years of mean follow-up. Our results are in line with those of previous studies that analyzed post-transplant recurrences of IgAN in kidney transplant recipients [5, 7, 13–15]. We show here that sIgA levels were higher in patients with IgAN compared to patients with other types of nephropathies. This is also in agreement with previous works. In one study, sIgA level was associated with pathological phenotypes. Thus, levels of sIgA were higher in patients with focal proliferative sclerosing IgAN, compared to patients with mild mesangial proliferative IgAN [16].

Many studies have analyzed the risk factors of IgAN recurrence in kidney transplant recipients. Potential risk factors related to recipients’ characteristics were younger age at transplantation [6], degree of proteinuria [7] and rapid progression of native IgA nephropathy [8]. Preformed donor IgA deposits have also been associated with an increased risk of IgAN recurrence after transplantation [10]. Moreover, some authors suggested that living donor graft could be a risk factor of post-transplant IgAN recurrence [6,9], but this observation has not been confirmed in other studies [14,17].

In the present work, we did not observe any significant difference between groups according to baseline characteristics, immunosuppressive regimen, cold ischemia time and delayed graft function rate. In addition, 6 months after transplantation, serum creatinine and proteinuria levels were not significantly different between groups. However, we observed that sIgA level was significantly higher in patients with IgAN recurrence as compared to patients without. We used ROC curves to analyze the predictive value of serum IgA, serum creatinine and proteinuria. Importantly, sIgA level was predictive of IgAN recurrence at both M6 and M12 post-transplant, while serum creatinine and proteinuria were not. Moreover, using univariate and multivariate analysis, sIgA was significantly associated with IgAN recurrence, whatever IgA was analyzed as a continuous or categorical variable and this association was independent of age and proteinuria level after successive adjustments. We finally analyzed survival free of IgAN recurrence according to the presence or not of sIgA level above to 222.5 mg/dL as determined by ROC analysis. By using this cut-off, survival free of IgAN recurrence was significantly higher in patients who had serum IgA level below 222.5 mg/dL, as compared to patients who had sIgA above 222.5 mg/dL.

These results are in part consistent with data from literature. Indeed, in a previous work, Zhang et al identified sIgA/C3 ratio as an independent risk factor for progression of IgAN in non-transplant patients [18]. In multivariate analysis, progression of IgAN was significantly predicted by serum IgA/C3 ratio [18]. Recently, Berthelot et al showed that pre-transplant serum levels of galactose-deficient IgA1 (Gd-IgA1) and IgA-IgG complexes were significantly higher in patients with IgAN recurrence compared to patients without or healthy subjects. Moreover, pre-transplant levels of IgA-soluble CD89 complexes were significantly lower in recurrent patients than in non-recurrent patients. Analysis of ROC curves confirmed the predictive values of galactose-deficient IgA1 and IgA-soluble CD89 complexes, suggesting that both markers could be predictive for IgAN recurrence after kidney transplantation [11]. In this work, pre-transplant IgA levels was not significantly different between patients with and without IgAN recurrence. In a more recent work, Berthoux et al also identified serum IgG autoantibodies towards galactose-deficient IgA1 at transplantation to be associated with a higher risk of IgAN recurrence [19]. In both studies, no longitudinal analysis of serum biomarkers was performed and none analyzed the predictive value of post-transplant IgA levels as we did in the present work.

Our work has several limitations. First, we did not perform systematic graft biopsies, which may have resulted in underestimation of IgAN recurrence. Next, the low number of patients with recurrence did not allow to analyze the relationship between sIgA levels and histological feature. Finally, the retrospective design and the intermediate number of IgAN patients in our cohort may have reduced the statistical power of the study. Besides these limitations, we still were able to identify a significant link between sIgA and post-transplant recurrence of IgAN. The concordance of our findings at M6 and M12 post-transplant also strengthens the results.

In conclusion, the present work confirmed the high prevalence of post-transplant recurrence of IgAN. Our results support a link between sIgA and the risk of post-transplant recurrence of IgAN. These results allow us to suggest that serum IgA level should be considered as a potential predictive factor of IgAN recurrence after kidney transplantation. **Disclosure:** The authors of this manuscript have no conflicts of interest to disclose.

## Supporting information

S1 Fig Flowchart of the study. (EPS)

S2 Fig Graft survival according to IgAN recurrence status. (EPS)

## Abbreviations

BMI: body mass index
CKD: chronic kidney disease
DBP: diastolic blood pressure
eGFR: estimated Glomerular Filtration Rate
Hb: hemoglobin
IgAN: IgA nephropathy
Ig: immunoglobulin
MDRD: Modification of Diet in Renal Disease equation
sAlb: serum albumin
SBP: systolic blood pressure
Scr: Serum creatinine
sIgA: serum IgA
